# OTUD7B Activates Wnt Signaling Pathway through the Interaction with LEF1

**DOI:** 10.3390/biom13061001

**Published:** 2023-06-16

**Authors:** Yuri Lee, Hai-long Piao, Jongchan Kim

**Affiliations:** 1 Department of Life Sciences, Sogang University, Seoul 04107, Republic of Korea; 2 CAS Key Laboratory of Separation Science for Analytical Chemistry, Dalian Institute of Chemical Physics, Chinese Academy of Sciences, Dalian 116023, China

**Keywords:** Wnt signaling, LEF1, OTUD7B, β-catenin

## Abstract

The Wnt signaling pathway plays a critical role in regulating normal cellular processes, including proliferation, differentiation, and apoptosis. Dysregulation of Wnt signaling has been implicated in various human diseases, including cancer. β-catenin and LEF1 are key mediators of Wnt signaling, and their dysregulation is a hallmark of many cancer types. In this study, we aimed to identify the deubiquitinases (DUBs) that regulate the Wnt signaling pathway through the essential component LEF1. Screening candidate DUBs from the human DUB library, we discovered that OTUD7B interacts with LEF1 and activates Wnt signaling. OTUD7B and LEF1 interact with each other through the UBA and HMG domains, respectively. Furthermore, OTUD7B promotes the nuclear localization of LEF1, leading to an increased interaction with β-catenin in the nucleus while not noticeably affecting ubiquitination on LEF1. Using qPCR array analysis, we found that OTUD7B overexpression leads to an upregulation of 75% of the tested Wnt target genes compared to the control. These findings suggest that OTUD7B may serve as a potential therapeutic target in human diseases, including cancers where Wnt signaling is frequently dysregulated.

## 1. Introduction

LEF1 (Lymphoid enhancer-binding factor 1) is a transcription factor that plays a crucial role in the development of multiple tissues, including the immune system, skin, and hair follicles [1,2,3]. It belongs to the TCF/LEF family of transcription factors, which are important regulators of Wnt signaling. While LEF1 is primarily recognized for its involvement in the development and differentiation of T cells, B cells, and natural killer cells [4,5,6,7], it is also known to participate in the regulation of the hair cycle and skin development [2,3]. LEF1 exerts its effects by binding to DNA through its high-mobility group (HMG) domain and interacting with other transcription factors and co-activators to control gene expression [8,9,10,11,12,13].

The Wnt signaling pathway is a complex network of signaling events that governs cellular processes such as cell proliferation, differentiation, and survival [14]. Abnormal Wnt signaling has been implicated in various types of cancer, including colorectal, breast, and liver cancer [15]. The canonical Wnt signaling pathway is activated when Wnt ligands bind to Frizzled receptors, leading to the stabilization and nuclear translocation of β-catenin in the nucleus, β-catenin interacts with TCF/LEF family of transcription factors to activate the transcription of target genes involved in cell growth and survival [14,15]. Dysregulation of β-catenin and LEF1 is a common feature in many types of cancer, highlighting the importance of understanding the underlying mechanisms of their regulation [15,16].

LEF1 activity is modulated by various post-translational modifications, including phosphorylation and ubiquitination [17,18,19,20]. Ubiquitination is a post-translational modification that involves the attachment of ubiquitin molecules to target proteins. Several E3 ubiquitin ligases have been reported to interact with LEF1 and promote its ubiquitination and degradation. For instance, the E3 ubiquitin ligase Pja2 has been shown to interact with LEF1 and regulate its protein levels through ubiquitination during stem cell differentiation [20]. Similarly, the E3 ubiquitin ligase NARF has been reported to interact with LEF1 and facilitate its degradation [19]. While multiple LEF1 E3 ligases have been identified, deubiquitinases (DUBs) for LEF1 have remained unknown until now.

In this study, we aimed to identify the DUBs that regulate Wnt signaling by screening a human DUB library and found OTUD7B as a LEF1-binding DUB. Surprisingly, OTUD7B did not exhibit deubiquitinating activity on LEF1 despite its interaction with LEF1. Instead, OTUD7B activated the Wnt signaling pathway, at least in part, by promoting the nuclear translocation of LEF1 and its interaction with β-catenin. Our data demonstrate the catalytic-independent function of a DUB in potentiating the Wnt signaling pathway. Further investigation is warranted to explore the potential of OTUD7B as a therapeutic target in human cancers by disrupting its interaction with LEF1 and inhibiting Wnt signaling pathway activation.

## 2. Materials and Methods

### 2.1. Cell Culture

The HEK293T cell line was obtained from the American Type Culture Collection and cultured in Dulbecco’s Modified Eagle Medium supplemented with 10% fetal bovine serum (FBS) and 1% penicillin-streptomycin. The cells were maintained in a 37 °C humidified incubator with 5% CO_2_.

### 2.2. Plasmids

The full-length LEF1 open reading frame (ORF) amplified from a human cDNA library was subcloned into the MYC-tagged expression vector using the gateway system (MYC-LEF1) (Invitrogen; Waltham, MA, USA). SFB-DUB vectors were constructed as previously described [21]. HA-ubiquitin, TOPflash, and FOPflash vectors were obtained from Addgene (Item #17608, #12456, and #12457, respectively; Watertown, MA, USA).

### 2.3. Reagents and Antibodies

Cycloheximide and MG132 were purchased from Cayman Chemical (Ann Arbor, MI, USA). Lithium chloride was obtained from Thermo Fisher Scientific (Waltham, MA, USA). S-protein agarose beads were obtained from Merck (Rahway, NJ, USA). MYC antibody-conjugated agarose beads were from Thermo Fisher Scientific (Waltham, MA, USA). The following antibodies were used: anti-FLAG (Proteintech; San Diego, CA, USA), anti-MYC (Proteintech; San Diego, CA, USA), anti-OTUD7B (Proteintech; San Diego, CA, USA), anti-cyclophilin B (Invitrogen; Waltham, MA, USA), anti-HSP90 (Santa Cruz Biotechnology; Santa Cruz, CA, USA), anti-HA (Santa Cruz Biotechnology; Santa Cruz, CA, USA), anti-V5 (Proteintech; San Diego, CA, USA), anti-α-tubulin (Proteintech; San Diego, CA, USA), and anti-lamin B1 (Santa Cruz Biotechnology; Santa Cruz, CA, USA).

### 2.4. Shor Interfering RNAs (siRNA)

siRNAs targeting human OTUD7B were purchased from GenePharma (Shanghai, China), and their sense sequences are provided as follows: siOTUD7B #1, 5′-UUCUUGAACGAUGUCAUCCTT-3′; siOTUD7B #2, 5′-CUUCUGUGUAUACCAGCCCTT-3′; siOTUD7B #3, 5′-AGGUCUCUCUCUAUGAAGCTT-3′.

### 2.5. Site-Directed Mutagenesis

MYC-LEF1 and SFB-OTUD7B truncation mutants were generated using the EZchange site-directed mutagenesis kit (Enzynomics; Daejeon, Korea) following the manufacturer’s protocol.

### 2.6. Immunoprecipitation and Pulldown Assays

For pulldown of SFB-DUBs, S-protein beads were utilized, and subsequent procedures including cell lysis, pulldown with S-protein beads, and immunoblotting were performed as described [21]. MYC-beads were used for immunoprecipitation of MYC-LEF1, and other steps were carried out following previously established protocols [21].

### 2.7. Deubiquitination Assay

HEK293T cells were co-transfected with MYC-LEF1, SFB-OTUD7B/SFB-GFP, and HA-ubiquitin constructs. Subsequent procedures were performed following the described protocol [21].

### 2.8. Cycloheximide Chase Assay

HEK293T cells were co-transfected with MYC-LEF1, MYC-GFP, and SFB-OTUD7B/SFB-GFP constructs. After 2 days, the cells were treated with cycloheximide (CHX) for the indicated durations. The cells were harvested, lysed with RIPA buffer using sonication, and subjected to immunoblotting.

### 2.9. Western Blotting

Western blotting assays following pulldown assays and immunoprecipitation were carried out as previously described [21].

### 2.10. Luciferase Assay

HEK293T cells were plated in quadruplicates in a 24-well plate. The cells were co-transfected with 150 ng of SFB-DUB/SFB-GFP, 100 ng of the firefly luciferase vector (TOPflash or FOPflash), and 1 ng of the Renilla luciferase vector. To co-transfect siRNAs and plasmid DNAs, the cells in the 24-cell plate were initially transfected with 0.17 µg of siOTUD7B #1, #2, #3 (total 0.5 µg) using X-tremeGENE siRNA transfection reagent (Roche; Basel, Switzerland). After 30 min, the cells were further transfected with 100 ng of firefly luciferase vector (TOPflash or FOPflash), and 1 ng of Renilla luciferase vector.

After 2 days, the cells were lysed, and luciferase activities were measured using the Dual-Luciferase Reporter Assay System (Promega; Madison, WI, USA) with the EnSpire Multimode Plate Reader (PerkinElmer; Waltham, MA, USA), following the manufacturer’s protocol. The firefly luciferase activity was normalized using the Renilla luciferase activity.

### 2.11. Cytoplasmic and Nuclear Fractionation

HEK293T cells were co-transfected with MYC-LEF1 and SFB-OTUD7B/SFB-GFP constructs. After 2 days, the cells were harvested, and cytoplasmic and nuclear proteins were extracted using the Nuclear Extraction Kit (EMD Millipore Corporation; Burlington, MA, USA). The proteins were quantified, and the protein samples were subjected to immunoblotting.

### 2.12. Pulldown Assay with Nuclear and Cytoplasmic Fractions

HEK293T cells were co-transfected with MYC-LEF1, SFB-β-catenin, and OTUD7B-V5/GFP-V5 (in pLX304 vectors) constructs. After 2 days, the cells were harvested. Prior to harvest, the cells were treated with 20 mM lithium chloride for 24 h. Cytoplasmic and nuclear lysates were extracted using the Nuclear Extraction Kit (EMD Millipore Corporation; Burlington MA, USA). Each lysate was pulled down with S-protein beads and immunoblotted with the indicated antibodies.

### 2.13. Quantitation of Immunoblot Images

The image processing software, ImageJ, was downloaded from https://imagej.nih.gov/ij/download.html (accessed on 17 February 2023) and utilized to quantify the immunoblotting band images. The nuclear and cytoplasmic levels of LEF1 were normalized using lamin B1 and α-tubulin levels, respectively.

### 2.14. Quantitative PCR Array

A qPCR screening kit for Human Wnt Signaling Targets was purchased from Bioneer (SH-0000-10) (Daejeon, Korea). HEK293T cells transfected with either SFB-GFP or SFB-OTUD7B were subjected to total RNA extraction using the MagListo RNA extraction kit (Bioneer, Daejeon, Korea) and RNase-free DNase I (Thermo Fisher Scientific. Waltham, MA, USA), followed by reverse transcription with the iScript cDNA synthesis kit (Bio-rad. Hercules, CA, USA). A total of 20 ng of cDNA was mixed with SYBR Green reagent (Enzynomics, Daejeon, Korea), and equal amounts were added to each well of a 96-well qPCR array plate containing gene-specific primer sets. Real-time PCR and data collection were performed using a CFX Connect instrument (Bio-rad. Hercules, CA, USA).

### 2.15. Statistical Analysis

The data are presented as mean ± s.e.m., and statistical significance was determined using the two-tailed unpaired *t*-test for comparing two independent groups. A *p*-value of less than 0.05 was considered significant.

## 3. Results

### 3.1. Identification of Human Deubiquitinases (DUBs) Interacting with LEF1

**Figure 1:**
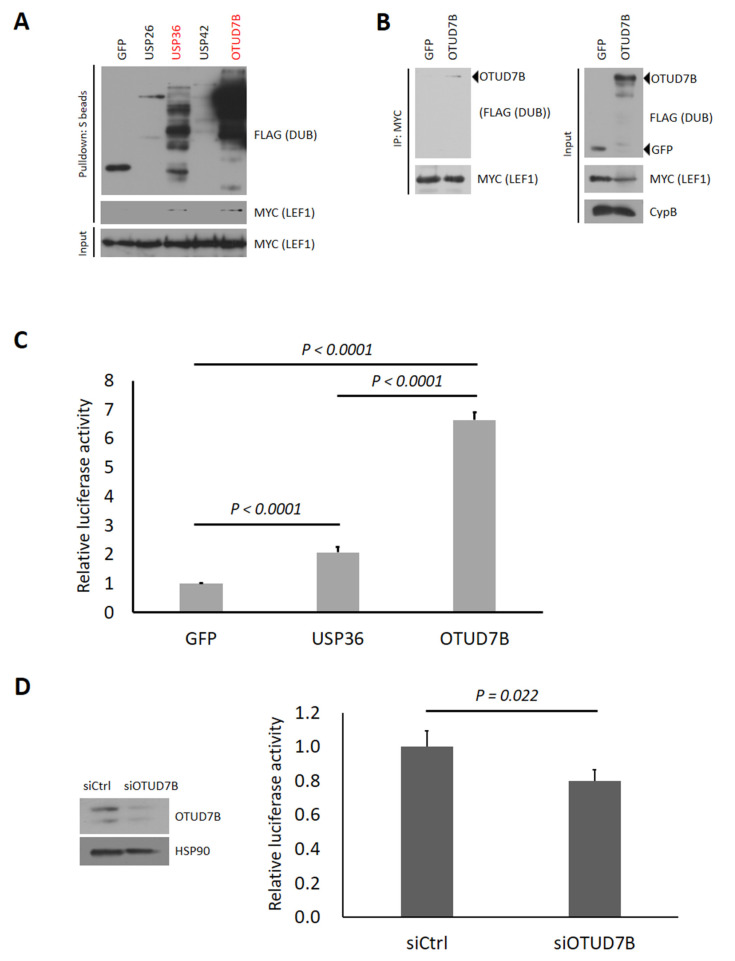
OTUD7B interacts with LEF1 and activates Wnt signaling. (**A**) HEK293T cells were transiently transfected with MYC-LEF1 and SFB-tagged DUBs (USP26, USP36, USP42, and OTUD7B) and subjected to pulldown using S-protein beads. The pulled-down DUBs and DUB-bound LEF1 were detected via immunoblotting using anti-FLAG and anti-MYC antibodies, respectively. (**B**) HEK293T cells were transiently transfected with MYC-LEF1 and SFB-OTUD7B and subjected to immunoprecipitation using MYC antibody-conjugated agarose beads. The pulled-down LEF1 and bound OTUD7B were detected via immunoblotting using anti-MYC and FLAG antibodies, respectively. (**C**) HEK293T cells were co-transfected with TOPflash (LEF1/β-catenin-responsive luciferase vector) or its mutant FOPflash, along with Renilla luciferase and SFB-USP36 or SFB-OTUD7B plasmids. Firefly luciferase activity from TOPflash and FOPflash was normalized to Renilla luciferase activity. The normalized TOPflash luminescence levels were further normalized to each normalized FOPflash luminescence. SFB-GFP was used as a negative control. (**D**) HEK293T cells were co-transfected with TOPflash or FOPflash, along with Renilla luciferase, and control siRNA or siRNA for OTUD7B. Left panel: the cells were subjected to lysis and immunoblotting was conducted using OTUD7B antibody to confirm the knockdown of endogenous OTUD7B. Right panel: Firefly luciferase activity from TOPflash and FOPflash was normalized to Renilla luciferase activity. The normalized TOPflash luminescence levels were further normalized to each normalized FOPflash luminescence. Control siRNA-transfected group was used as a negative control. Error bars represent the standard error of the mean (S.E.M.). Statistical significance was determined using an unpaired *t*-test.

Previously, we conducted luciferase reporter assays to screen human DUBs involved in regulating the Wnt signaling pathway [22]. In this experiment, we observed that six human DUBs, namely USP36, USP42, DUB3, OTUD7B, USP2, and USP26, significantly up-regulated Wnt signaling activity. Among these DUBs, USP2 and DUB3 were found to interact with β-catenin and deubiquitinate it. However, USP26 and USP42 did not interact with β-catenin, and although USP36 and OTUD7B interacted with β-catenin, they failed to deubiquitinate it. Based on these findings, we selected four DUBs (USP36, USP42, OTUD7B, and USP26) to investigate their interaction with LEF1 and their role in regulating Wnt signaling activity. To identify DUBs that interact with LEF1, we performed a pulldown assay using four SFB-tagged DUBs. Among the four DUBs tested, USP36 and OTUD7B were found to interact with MYC-LEF1 (Figure 1A).

### 3.2. OTUD7B Promotes and Its Depletion Suppresses the Transcriptional Activity of LEF1

We then examined which DUB has a greater effect on the transcriptional activity of LEF1. The luciferase reporter containing wildtype TCF/LEF binding sites (TOPflash) and mutated TCF/LEF binding sites (FOPflash) was transfected with SFB-USP36 or SFB-OTUD7B. Compared to FOPflash luciferase levels, both USP36 and OTUD7B increased the luciferase activities (TOPflash), but OTUD7B significantly enhanced LEF1 activity to a greater extent (6.64-fold) than USP36 did (2.04-fold) (Figure 1C). In addition, we transiently depleted OTUD7B expression using siRNAs and assessed the impact on the transcriptional activity of LEF1. Figure 1D demonstrates that the loss of OTUD7B significantly suppressed LEF1 activity. Therefore, we selected OTUD7B as the LEF1-interacting and LEF1-activating DUB and the reverse co-immunoprecipitation result confirmed their physical interaction once again (Figure 1B). Building upon these findings, we proceeded to investigate the mechanisms through which OTUD7B regulates LEF1 to activate the Wnt signaling pathway.

### 3.3. LEF1 Interacts with OTUD7B through the HMG and UBA Domains, Respectively

**Figure 2:**
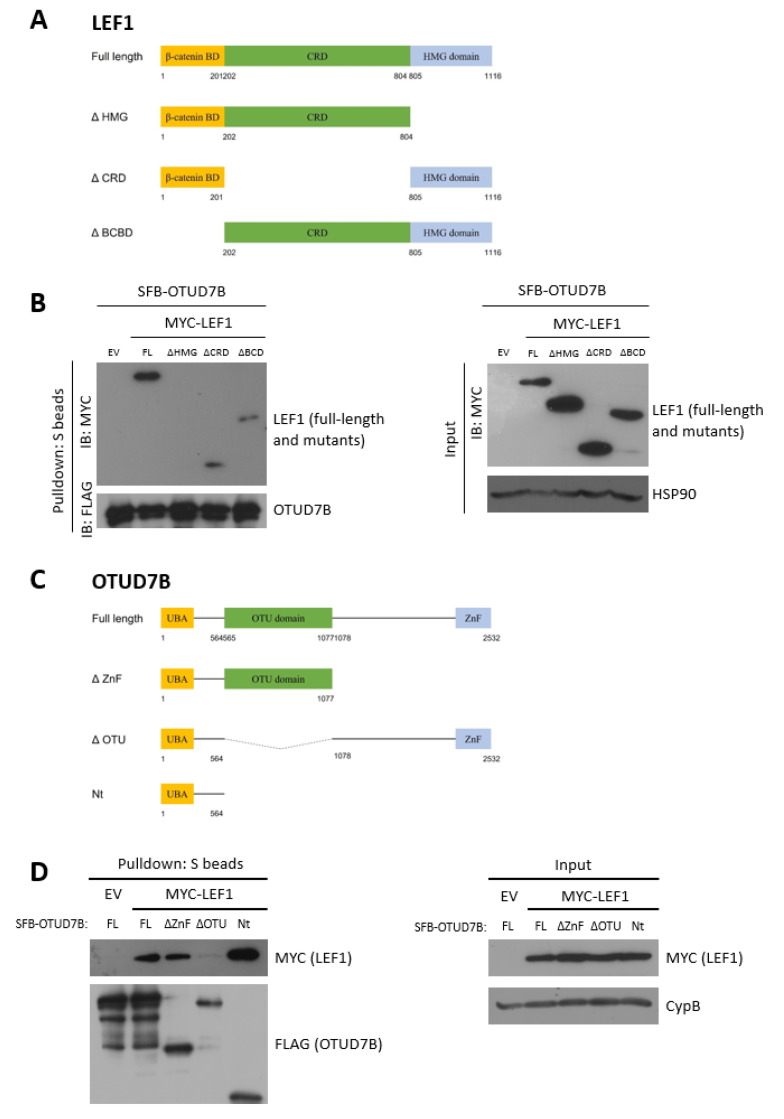
HMG domain of LEF1 and UBA domain of OTUD7B are necessary for their interaction. (**A**) Schematic representation of full-length LEF1 and its deletion mutants. ΔBCBD: β-catenin binding domain deleted; ΔCRD: context-dependent regulatory domain deleted; ΔHMG: high mobility group DNA binding domain deleted. (**B**) Full-length OTUD7B was co-transfected with full-length LEF1 or its deletion mutants into HEK293T cells. OTUD7B was pulled down using S-protein beads, and LEF1, its mutants, and OTUD7B were detected using anti-MYC and anti-FLAG antibodies, respectively. Heat shock protein 90 (HSP90) was used as the loading control. EV: empty vector; FL: full-length LEF1. (**C**) Schematic representation of full-length OTUD7B and its deletion mutants. ΔZnF: zinc finger domain deleted; ΔOTU: ovarian tumor domain deleted; Nt: amino-terminus domain; UBA: ubiquitin-associated domain. (**D**) Full-length LEF1 was co-transfected with full-length OTUD7B or its deletion mutants into HEK293T cells. OTUD7B and its mutants were pulled down using S-protein beads, and LEF1, OTUD7B, and its mutants were detected using anti-MYC and anti-FLAG antibodies, respectively. Cyclophilin B (CypB) was used as the loading control. EV: empty vector; FL: full-length OTUD7B.

To determine which domains are responsible for the interaction between LEF1 and OTUD7B, we generated three deletion mutants of LEF1: LEF1ΔHMG (High Mobility Group domain deleted, aa 1-804), LEF1ΔCRD (context-dependent regulatory domain deleted, aa 1-201 & 805-1116), and LEF1ΔBCBD (β-catenin binding domain deleted, aa 202-1116) (Figure 2A) using the MYC-LEF1 plasmid. We co-transfected these constructs and full-length SFB-OTUD7B into HEK293T cells and pulled down SFB-OTUD7B with S-protein agarose beads. As shown in Figure 2B, LEF1ΔCRD and LEF1ΔBCD interacted with OTUD7B, but LEF1ΔHMG did not. This result demonstrates that OTUD7B binds to LEF1 through its HMG domain. The HMG domain is a DNA-binding motif shared by many non-histone chromatin-associated proteins and transcription factors [23,24].

Next, we generated three deletion mutants of OTUD7B: OTUD7BΔZnF (Zinc Finger domain deleted, aa 1-1077), OTUD7BΔOTU (Ovarian Tumor domain deleted, aa 1-564 & 1078-2532), and OTUD7BNt (UBA domain-containing, 1-564) using the SFB-OTUD7B plasmid (Figure 2C) and attempted to identify the LEF1-binding region on OTUD7B. Similarly, we co-transfected these constructs and full-length MYC-LEF1 into HEK293T cells and pulled down full-length and mutant SFB-OTUD7B with S-protein agarose beads. As shown in Figure 2D, the full-length OTUD7B and all three mutant forms interacted with LEF1. This result suggests that LEF1 binds to OTUD7B through its N-terminus, which contains the UBA domain. Proteins with a UBA (Ubiquitin-associated) domain non-covalently interact with ubiquitin [25].

### 3.4. OTUD7B Does Not Deubiquitinate nor Stabilize LEF1

**Figure 3:**
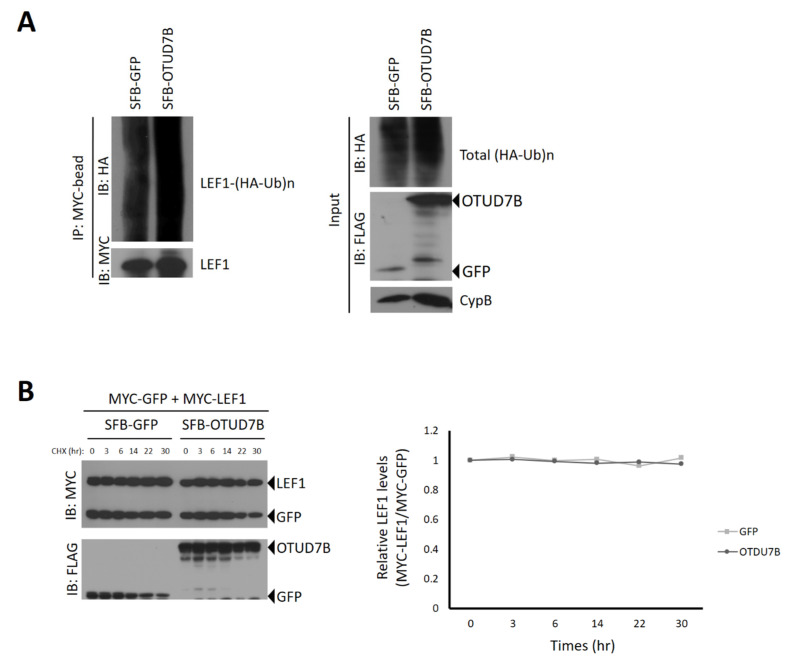
OTUD7B does not deubiquitinate LEF1 and does not affect its protein stability. (**A**) HEK293T cells were co-transfected with SFB-GFP or SFB-OTUD7B, MYC-LEF1, and HA-ubiquitin plasmids. After treating the cells with MG132 (10 μM) for 6 h, cell lysates were obtained and LEF1 was pulled down using MYC antibody-conjugated agarose beads. Immunoblotting was performed using antibodies against HA (to detect polyubiquitinated LEF1) and MYC (to detect LEF1). (**B**) HEK293T cells were co-transfected with SFB-OTUD7B or SFB-GFP, MYC-GFP, and MYC-LEF1 plasmids. The cells were treated with the translation inhibitor cycloheximide (100 μg/mL) for the indicated time periods. The protein stability of MYC-LEF1 was examined using anti-MYC antibody. The relative protein levels of MYC-LEF1 were quantified by normalizing to the expression levels of MYC-GFP control.

Since LEF1 is targeted for proteasomal degradation through ubiquitination by multiple E3 ligases, including NARF and Pja2 [19,20], we investigated whether OTUD7B deubiquitinates LEF1 and regulates its protein stability. As shown in Figure 3, OTUD7B did not noticeably deubiquitinate LEF1, and its protein stability was not affected by OTUD7B. This suggests that the deubiquitinase activity of OTUD7B may not be necessary for regulating LEF1′s transcriptional activity. Furthermore, the protein stability of LEF1 did not show significant changes during the 30-h period tested in this study. Therefore, we speculate that OTUD7B may influence the transcriptional activity of LEF1 in an enzyme activity-independent manner.

### 3.5. OTUD7B Promotes the Nuclear Translocation of LEF1

**Figure 4:**
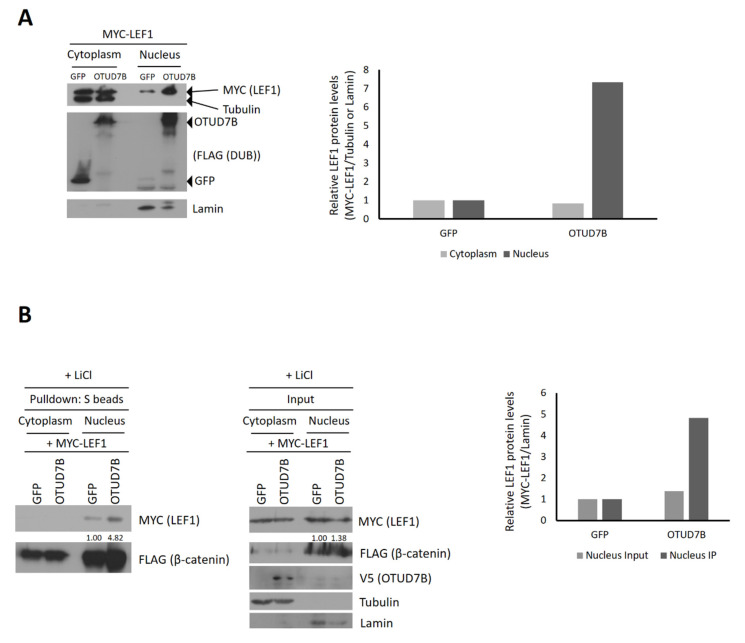
OTUD7B promotes nuclear translocation of LEF1 and its interaction with β-catenin in the nucleus. (**A**) HEK293T cells were transiently transfected with MYC-LEF1 and either SFB-GFP or SFB-OTUD7B plasmids. Cytoplasmic and nuclear fractions of the cell lysates were separated and analyzed via immunoblotting using anti-MYC and anti-FLAG antibodies to detect LEF1 and OTUD7B proteins, respectively. Anti-α-tubulin and anti-lamin B1 antibodies were used as loading controls for the cytoplasmic and nuclear fractions, respectively. Left panel: Immunoblotting analysis of cytoplasmic and nuclear fractions. Right panel: Quantification of LEF1 protein levels, with cytoplasmic MYC-LEF1 normalized to α-tubulin and nuclear MYC-LEF1 normalized to lamin B1. (**B**) HEK293T cells were co-transfected with MYC-LEF1, SFB-β-catenin, and either GFP-V5 or OTUD7B-V5. Prior to cell lysis, the cells were treated with 20 mM lithium chloride. β-catenin was pulled down using S-protein agarose beads, followed by immunoblotting with anti-MYC (to detect LEF1), anti-FLAG (to detect β-catenin), and anti-V5 (to detect OTUD7B) antibodies. Anti-α-tubulin and anti-lamin B1 antibodies were used to detect loading controls for the cytoplasmic and nuclear fractions, respectively. Left panels: Immunoblotting analysis following the pulldown assay of cytoplasmic and nuclear fractions. Right panel: Quantification of β-catenin-bound and total LEF1 protein levels in the nucleus, with nuclear MYC-LEF1 normalized to lamin B1.

We hypothesized that OTUD7B may be involved in the subcellular localization of LEF1 if OTUD7B does not affect protein stability. To investigate this, we co-transfected MYC-LEF1 with SFB-OTUD7B or SFB-GFP plasmids into HEK293T cells and separated the cytoplasmic and nuclear fractions of the cell lysates. The protein levels of LEF1 in both fractions were compared via immunoblotting using an anti-MYC antibody. Surprisingly, when OTUD7B was overexpressed, LEF1 was significantly more abundant in the nuclear fraction compared to the cytoplasmic fraction (Figure 4A). This indicates that OTUD7B does not affect LEF1 levels in the cytoplasm (1.00 in GFP control vs. 0.84 in OTUD7B), but strongly promotes its nuclear localization (1.00 in GFP control vs. 7.33 in OTUD7B). These findings suggest that the interaction with OTUD7B facilitates the nuclear translocation of LEF1, resulting in enhanced transcriptional activity.

### 3.6. OTUD7B Promotes the Interaction between LEF1 and β-Catenin

We further investigated whether the increased nuclear translocation of LEF1 by OTUD7B leads to enhanced transcriptional activity by promoting the interaction between LEF1 and β-catenin. For this purpose, we performed a pulldown assay using nuclear and cytoplasmic fractions of cell lysates. HEK293T cells were transiently transfected with MYC-LEF1, SFB-β-catenin, and V5-tagged GFP or OTUD7B, and prior to cell lysis, the cells were treated with lithium chloride to increase the availability of nuclear β-catenin. β-catenin was pulled down using S-protein agarose beads, and the bound LEF1 was assessed in both fractions via immunoblotting. As shown in Figure 4B, OTUD7B induced a 4.82-fold increase in the β-catenin-bound LEF1 levels in the nucleus, while the nuclear LEF1 levels were only increased by 38% at this time. This clearly demonstrates that OTUD7B promotes the nuclear localization of LEF1 and enhances the interaction between LEF1 and β-catenin in the nucleus, resulting in enhanced transcriptional activation.

### 3.7. OTUD7B Upregulates the Expression of Wnt Target Genes

**Figure 5:**
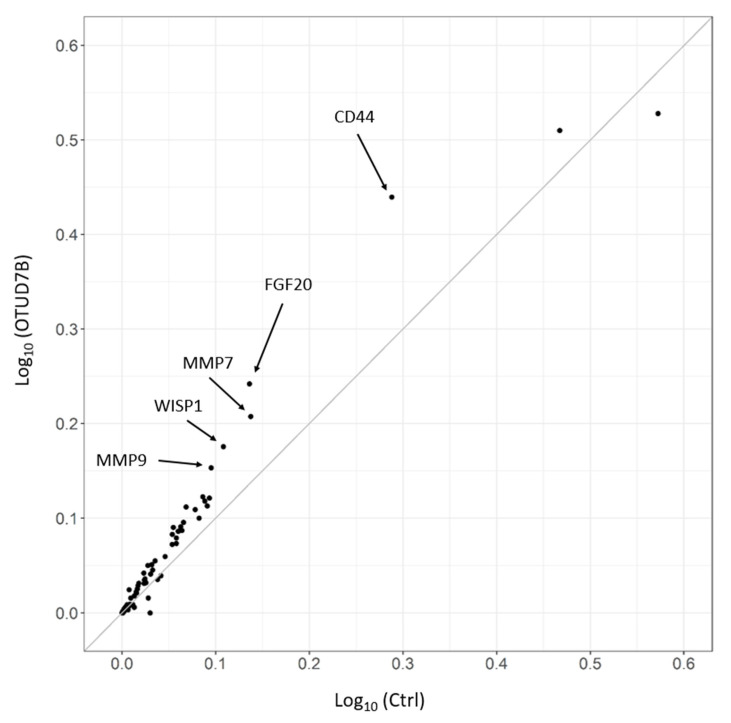
OTUD7B activates the expression of Wnt signaling target genes. The scatterplot depicts the quantitative PCR array results of Wnt signaling target genes. Gene expression levels were compared between cells transfected with SFB-OTUD7B and cells transfected with SFB-GFP (control). Dots positioned above and below the diagonal represent upregulated and downregulated genes by OTUD7B, respectively. Arrows indicate the five most upregulated Wnt target genes.

LEF1 is a crucial regulator of the canonical Wnt signaling pathway. Therefore, we investigated whether OTUD7B plays a role in Wnt signaling activation by promoting the nuclear localization of LEF1 and its interaction with β-catenin. To address this question, we utilized a quantitative PCR array kit that profiles the expression of 84 Wnt target genes and examined whether OTUD7B enhances the overall expression of these genes. We prepared cDNA samples from HEK293T cells transiently transfected with either SFB-GFP (control) or SFB-OTUD7B, and performed qPCR analysis. Our results revealed that 63 out of 84 genes were upregulated by OTUD7B (Figure 5), including CD44, FGF20, MMP7/9, and WISP1. This finding clearly demonstrates that OTUD7B mediates the activation of Wnt target gene expression.

## 4. Discussion

OTUD7B/Cezanne, a member of the ovarian tumor (OTU) domain-containing family of deubiquitinases (DUBs), is a critical regulator of various cellular processes, including DNA damage response and immune regulation [26,27,28,29,30]. Dysregulation of OTUD7B has been observed in several types of cancer, such as lung, breast, liver, and pancreatic cancers. In these cancers, the upregulation of OTUD7B promotes the stabilization and activation of TRAF3, estrogen receptor α, and N1ICD, leading to uncontrolled cell growth and tumor formation [31,32,33,34,35,36]. Conversely, downregulation of OTUD7B has been associated with impaired DNA damage response and increased genomic instability [29,37].

In our previous study, we discovered OTUD7B as one of the DUBs that activate the Wnt signaling pathway. While OTUD7B interacted with β-catenin, it did not deubiquitinate β-catenin [22]. This finding prompted us to investigate OTUD7B’s role in regulating the transcriptional activity of LEF1. Despite its interaction with LEF1 and enhancement of Wnt signaling activity, it was surprising that OTUD7B did not deubiquitinate LEF1 or affect its protein stability. As a result, we hypothesized that OTUD7B promotes Wnt signaling by facilitating the nuclear localization of LEF1 and/or its interaction with β-catenin within the nucleus. Notably, our research revealed that OTUD7B induces the translocation of LEF1 to the nucleus and facilitates its interaction with β-catenin in the nuclear compartment. The interaction between LEF1 and OTUD7B occurs through the HMG and UBA domains of LEF1 and OTUD7B, respectively. Furthermore, the catalytic-independent function of OTUD7B was found to activate the expression of 75% of Wnt signaling target genes.

Catalytic-independent functions are often observed in various kinases and DUBs. For example, the receptor tyrosine kinase EGFR interacts with the proapoptotic molecule PUMA in the cytoplasm, leading to tumor drug resistance. EGFR also interacts with certain transcription factors in the nucleus to regulate target gene expression [38,39]. RAF1 inhibits several kinases, including ROK-α, ASK1, and MST2, through protein-protein interactions rather than phosphorylation [40,41]. Similarly, DUBs have been shown to exert catalytic-independent functions. For instance, OTUB1 inhibits several E2 ubiquitin-conjugating enzymes through protein-protein interactions [42,43,44], and OTULIN interferes with endosomal trafficking by interacting with SNX27, a protein involved in protein trafficking and endocytosis of plasma membrane receptors [45].

LEF1 is known to be transported to the nucleus by importin α, which recognizes the nuclear localization sequence located in the HMG domain of LEF1 [46]. Since OTUD7B also binds to the HMG domain of LEF1, their interaction may affect the function of importin α, facilitating the nuclear localization of LEF1.

Interestingly, OTUD7B has been shown to deubiquitinate β-catenin in vascular smooth cells [47], although it failed to do so in our model cell line, HEK293T [22]. This suggests that OTUD7B-driven deubiquitination may be cell-context-dependent. Additionally, since LEF1 and β-catenin interact as two essential transcriptional components of the Wnt signaling pathway, OTUD7B may facilitate the nuclear localization of both LEF1 and β-catenin as a protein complex. This may enhance the overall expression of Wnt signaling target genes, as observed in our present study.

Studies have exhibited that OTUD7B stimulates cancer progression in multiple human cancers [35,36,48,49]. This finding implicates that OTUD7B may serve as a potential therapeutic target for cancer treatment. However, caution needs to be applied when developing and using OTUD7B inhibitors, especially for cancers where the Wnt signaling pathway is dysregulated. The reason for caution lies in the catalytic-independent function of OTUD7B in activating LEF1. Inhibiting the enzymatic activity of OTUD7B may not efficiently suppress Wnt signaling. Instead, a more effective approach could involve the use of small peptides that interfere with the interaction between LEF1/β-catenin and OTUD7B. Such peptides may display better signaling inhibition.

## Data Availability

Not applicable.
